# Improving the use of environmental diversity as a surrogate for species representation

**DOI:** 10.1002/ece3.3651

**Published:** 2017-12-10

**Authors:** Fabio Albuquerque, Paul Beier

**Affiliations:** ^1^ Science and Mathematics Faculty College of Integrative Sciences and Arts Arizona State University Mesa AZ USA; ^2^ School of Forestry Northern Arizona University Flagstaff AZ USA

**Keywords:** biodiversity, conservation, maxdisp, p‐median, spatial prioritization

## Abstract

The continuous p‐median approach to environmental diversity (ED) is a reliable way to identify sites that efficiently represent species. A recently developed maximum dispersion (maxdisp) approach to ED is computationally simpler, does not require the user to reduce environmental space to two dimensions, and performed better than continuous p‐median for datasets of South African animals. We tested whether maxdisp performs as well as continuous p‐median for 12 datasets that included plants and other continents, and whether particular types of environmental variables produced consistently better models of ED. We selected 12 species inventories and atlases to span a broad range of taxa (plants, birds, mammals, reptiles, and amphibians), spatial extents, and resolutions. For each dataset, we used continuous p‐median ED and maxdisp ED in combination with five sets of environmental variables (five combinations of temperature, precipitation, insolation, NDVI, and topographic variables) to select environmentally diverse sites. We used the species accumulation index (SAI) to evaluate the efficiency of ED in representing species for each approach and set of environmental variables. Maxdisp ED represented species better than continuous p‐median ED in five of 12 biodiversity datasets, and about the same for the other seven biodiversity datasets. Efficiency of ED also varied with type of variables used to define environmental space, but no particular combination of variables consistently performed best. We conclude that maxdisp ED performs at least as well as continuous p‐median ED, and has the advantage of faster and simpler computation. Surprisingly, using all 38 environmental variables was not consistently better than using subsets of variables, nor did any subset emerge as consistently best or worst; further work is needed to identify the best variables to define environmental space. Results can help ecologists and conservationists select sites for species representation and assist in conservation planning.

## 1 INTRODUCTION

Because of the lack of reliable biological data for conservation planning, surrogates are often used to represent aspects of biodiversity (Moilanen, Wilson, & Possingham, 2009). Surrogates are well‐mapped taxonomic (e.g., occurrences of a given taxon, umbrella species, focal species) or environmental features (e.g., land‐forms, soil types, vegetation types, NDVI) in the planning region.

The environmental diversity (ED) approach, proposed by Faith and Walker (1996), is a rarely used surrogate based on environmental dissimilarities in a continuous (unbinned) environmental space quantified as an ordination (Faith & Walker, 1996; Faith, Ferrier, & Walker, 2004). This framework assumes that species have a unimodal niche in environmental space (Faith et al., 2004), such that larger distances between sites in environmental space imply greater species‐composition differences (Faith & Walker, 1996). A low‐cost surrogate‐like ED could be useful for conservation planning because no region of the world has high‐resolution information on the distribution of species.

Environmental diversity uses the *p*‐median selection algorithm to select sites to span the underlying environmental space as quantified in the ordination. The *p*‐median algorithm was initially proposed to specify the best set of facilities or services locations (e.g., hospitals, fire stations) that minimize the average travel distance to the locations (demand points) in a planning area (Faith & Walker, 1996; Reese, 2006). In the ED context, the space is a multidimensional environmental space (not geographical space) representing the range of environmental conditions, and the demand points are distributed uniformly throughout the environmental space (Faith, 2003). The continuous p‐median algorithm selects *p* sites that minimize the sum of distances (in environmental space) from each “demand point” to the nearest selected site. The idea is that a set of *p* sites that collectively represent environmental diversity will efficiently represent diversity of many components of biodiversity (e.g., species) whose true distributions are not known. The ordination space is limited to 2 or 3 dimensions because p‐median calculations become computationally prohibitive in higher dimensional space.

Recently, Engelbrecht, Robertson, Stoltz, and Joubert (2016) suggested a new approach to ED, which they called the greedy maximum dispersion or maxdisp approach. Maxdisp differs from p‐median by minimizing the sum of inverse squared distances between selected sites, instead of minimizing the sum of distances between selected sites and demand points, to produce an even spread of sites through environmental space. Because demand points are not needed, there is no need to collapse environmental space to two dimensions—maxdisp can operate in full‐dimensional PCA space, or even in space defined by the raw environmental data. Engelbrecht et al. (2016) reported that maxdisp performed as well as continuous p‐median for four empirical datasets, one modeled dataset, and one simulated set of biodiversity data in South Africa. The maxdisp approach is computationally simple, even in high‐dimensional ordination or raw environmental space. Thus, if maxdisp performs as well as continuous p‐median in many settings, it could be a better optimization approach.

The choice of environmental variables could also affect the efficiency of ED. In general, we expected that including all environmental variables known to affect species distributions would produce a better ED model. On the other hand, including an irrelevant variable could introduce noise that would decrease the complementarity value of environmentally diverse sites. And although climate is certainly a driver of complementarity, the crude interpolation of climate variables (lat‐long interpolation with a single elevation covariate) could introduce errors. Moreover, some types of variables may better define the environmental space for a particular taxon, spatial extent, or spatial resolution.

Herein, we compare maxdisp ED to continuous p‐median ED for 12 sets of biodiversity data (Table 1) and investigate whether the types of variables used to define environmental space (e.g., climate, topographic, and insolation variables) affect the ability of ED to select sites that represent species efficiently. Our objective was to determine if the maxdisp approach is as effective a surrogate as continuous p‐median approach to ED. If maxdisp performed well in our tests, the result would justify further work to improve the ED approach so that it can be used to prioritize sites for conservation.

**Table 1:** Datasets used to evaluate environment diversity as a surrogate to meet the goal of species representation

Taxon, geographic area	No. of cells or sites	Size of grid cell or inventory site	No. of species	Type of dataset^a^	Source
Plants, Sierra Nevada, Spain	595	200 m	255	Inventory	SNGCO (2013)
Birds, Arizona, USA	1,317	2.4 km	359	Inventory	Corman and Wise‐Gervais (2005)
Plants, UK	2,242	10 km	1,456	Atlas	Preston, Pearman, and Dines (2002)
Birds, Spain	5,301	10 km	294	Atlas	INB (2007)
Plants, Botswana	556	25 km	2,237	Atlas	PRECIS (2014)
Plants, Namibia	998	25 km	3,566	Atlas	PRECIS (2014)
Plants, Zimbabwe	360	25 km	1,338	Atlas	PRECIS (2014)
Vertebrates, Western Europe	2,195	50 km	771	Atlas	
Amphibians	2,195	50 km	55	Atlas	Gasc et al. (1997)
Reptiles	2,195	50 km	106	Atlas	Gasc et al. (1997)
Birds	2,195	50 km	471	Atlas	Mitchell‐Jones et al. (1999)
Mammals	2,195	50 km	142	Atlas	Hagemeijer and Blair (1997)

In each “inventory” dataset, the sites were a systematic subsample of the geographic area of interest, and an attempt was made to inventory a representative set of sites in the study area. In each “atlas” dataset, each site was a grid cell, survey efforts did not cover the entirety of each grid cell, and the sites collectively comprised the entire geographic area of interest.

## 2 METHODS

### 2.1 Species dataset

We selected 12 biodiversity inventories and atlases that span a broad range of taxa (plants, birds, mammals, reptiles, and amphibians), spatial extents, and resolutions (Table 1). We considered the sites in the dataset to be the entire planning area, even though in some cases (e.g., plants of Zimbabwe, birds of Arizona) species data did not cover all sites within the boundaries of the study region.

### 2.2 Environmental datasets

For all sites within each dataset, we obtained 38 potential predictor variables available for all regions of the world (Table S1). We obtained temperature and precipitation variables from Hijmans, Cameron, Parra, Jones, and Jarvis (2005), insolation variables from Neteler (2005), NDVI (normalized difference vegetation index) from Tucker, Pinzon, and Brown (2004), and elevation and slope from USGS (2014). We calculated topographic diversity and aspect diversity (Benito, Cayuela, & Albuquerque, 2013) from elevation data. We calculated the mean, maximum, minimum, or range of each environmental variable across each grid cell. We selected these variables because they were available for all datasets, and each has been shown to be associated with site complementarity or species richness (Stein, Gerstner, & Kreft, 2014 and references therein).

### 2.3 Types of environmental variables

We applied continuous p‐median and maxdisp to five characterizations of environmental space, each defined by a particular combination of types of environmental variables (Table S1). The first combination of variable types (CVT1) included all 38 environmental variables; CVT2 included climate (temperature and precipitation) and NDVI variables; CVT3 included the climate and topographic variables; CVT4 consisted of only the climate variables; and CVT5 comprised the insolation and topographic variables. We tested whether the environmentally diverse sites selected using any particular CVT consistently represented more species than sites selected using other CVTs.

### 2.4 Defining environmental space

We used principal components analysis to reduce multicollinearity, which could distort environmental space. We used the Kaiser criterion to identify factors with eigenvalues >1.0 (Kaiser 1960). The number of significant PCA factors for each geographical area and CVT varied from two (Sierra Nevada) to eight (Spain and Botswana), with a mode of four factors (Table S2).

For p‐median ED, we then used nonmetric multidimensional scaling (NMDS) (Minchin, 1987) to create a two‐dimensional ordination space where intersite distance reflects distances in full PCA space. Reduction in two dimensions was required to create an array of hypothetical demand points for the continuous p‐median algorithm in POPSTAR (Resende & Werneck, 2003). For Maxdisp, we calculated Euclidean distances in space defined by the significant PCA factors. Ordinations were performed using R package vegan (R Core Team, 2014, Oksanen, Blanchet, Kindt, Legendre, & Minchin, 2013).

### 2.5 Computation of the ED metrics

Environmental diversity models were constructed for five CVTs. We used the continuous p‐median ED and maxdisp ED approaches to select environmentally diverse sites.

In the continuous p‐median ED approach, we first divided each NMDS axis into equal intervals to create a 50 × 50 grid in two‐dimensional NMDS ordination space and used the 2,500 grid cell centroids as demand points. (We ignore the discrete p‐median approach because it is inferior to continuous p‐median on both theoretical grounds (Faith, 2003, 2011) and empirical tests, Beier & Albuquerque, 2015; Engelbrecht et al., 2016). We then used the hybrid heuristic p‐median procedure in POPSTAR with 32 random starts, and hybridization among the top 10 solutions to identify a solution that best spanned ordination space. We performed five runs per dataset and CVT, varying *p* to select 15%, 20%, 25%, 30%, and 35% of the total number of sites in each dataset.

In the maxdisp model, we first used significant PCA factors to produce a Euclidean distance matrix among planning sites (grid cells). We then minimized the inverse of the square of distances between selected sites to select sites evenly distributed in environmental space (Engelbrecht et al., 2016):

$$\begin{array}{cc} & \text{minimize}\;\sum_{i=1}^{n-1}\sum_{j=i+1}^{n}\frac{1}{d_{\mathrm{ij}}^{2}}\\ & \text{Subject to:}\\ & \sum_{i}^{n}x_{i}=m,x_{i}\in \left\{0,1\right\} \end{array}$$

where *n* is the number of sites (planning units) available for starting conservation actions, *i* and *j* are indices for site, *x* is the control variable indicating if a site has been selected or not, and *m* is the set of sites that must be selected. We used the “greedy” maxdisp heuristic (which at each step adds the site that minimizes the function) instead of an “interchange” heuristic (which swaps out large sets of sites at each step), because Engelbrecht et al. (2016) reported that the greedy heuristic performed as well as the interchange heuristic, and mimics the situation in which a decision‐maker adds one site at a time to the set of protected sites.

The maxdisp algorithm is a modified version of the maximum diversity algorithm—a metaheuristic algorithm used to select a set of *m* elements from a set of *n* elements in the way to maximize the sum of distances between the chosen elements (Engelbrecht et al., 2016).

### 2.6 Assessing effectiveness of ED metrics as a surrogate

We used the species accumulation index, SAI (Rodrigues & Brooks, 2007), to evaluate the efficiency of ED (continuous and maxdisp) in representing each taxon and to assess if the choice of variables and environmental scenarios influence the efficiency of ED as a surrogate for species representation. SAI relates the number of species represented in the set of sites selected using the surrogate (***S***), to the largest number of species that can be represented in the same number of sites (an optimum value ***O***) and to number of species represented in the same number of randomly selected sites (***R***). We used the core area version of Zonation (Moilanen et al., 2014) to calculate ***O***. Zonation is a reverse stepwise heuristic algorithm that produces a hierarchic ranking of conservation priority for all sites throughout the landscape (Moilanen et al., 2009). To calculate *R*, we randomly selected sets of p sites and calculated the number of species represented at least once in the randomly selected cells. We repeated the random selection procedure 1,000 times, and used the mean *R* value.

Formally, SAI = (*S*–*R*)/(*O*–*R*). SAI is scaled −∞ to 1; a positive SAI is a measure of efficiency whereas negative SAI indicates a worse than random result, and 0 indicates random performance. For example, SAI of 0.5 indicates that the surrogate was 50% as effective as the optimal solution in its ability to improve on random selection of sites. We calculated SAI at 15%, 20%, 25%, 30%, and 35% of the landscape hypothetically reserved and used the median of these 5 SAI values as an overall estimate of surrogate performance.

## 3 RESULTS

Sites selected by both approaches to ED represented more species than occurred in randomly selected sites for most datasets and combinations of environmental variables (Table 2). Continuous ED represented slightly fewer species than randomly selected sites for plants of UK (SAI < 0 for each of the five sets of environmental variables). Maxdisp ED represented substantially more species (maximum and median SAI 0.20 higher) than continuous p‐median ED for five datasets (plants of UK, plants of Zimbabwe, reptiles of Europe, birds of Europe, and combined vertebrates of Europe). For the other seven datasets, the two ED approaches performed approximately equally (Table 2). Although continuous p‐median ED represented mammals of Europe at 100% efficiency for two sets of environmental variables, it represented mammals of Europe poorly (negative SAI) for the other three sets of environmental variables.

**Table 2:** Efficiency (Species Accumulation Index [SAI] values^a^) of two approaches to environmental diversity (ED), namely continuous p‐median and maxdisp, for each of 12 biodiversity datasets and five sets of variables used to define environmental space

Biodiversity dataset	ED approach	Types of variables used to define environmental space				
		All 38 variables	Climate, insolation, topography	Climate, NDVI	Climate only	Insolation, topography
Plants, Sierra Nevada	p‐median	0.00	−0.01	−0.03	**0.10**	0.06
	maxdisp	−0.03	0.01	−0.04	−0.05	**0.15**
Birds, Arizona	p‐median	0.29	−0.13	**0.39**	−0.09	−0.08
	maxdisp	0.08	−0.08	**0.22**	0.05	−0.18
Plants, UK	p‐median	−**0.01**	−0.14	−0.20	−0.07	−0.18
	**maxdisp**	0.25	−0.14	**0.41**	0.22	−0.09
Birds, Spain	p‐median	−0.14	0.39	**0.55**	0.33	−0.06
	maxdisp	0.52	0.34	**0.75**	0.19	0.15
Plants, Namibia	p‐median	**0.38**	0.08	0.13	0.15	0.06
	maxdisp	0.25	**0.28**	0.24	0.25	0.24
Plants, Botswana	p‐median	0.16	0.22	0.10	**0.31**	0.25
	maxdisp	0.38	**0.38**	0.35	0.36	0.23
Plants, Zimbabwe	p‐median	0.37	0.34	**0.41**	0.35	−0.19
	**maxdisp**	0.57	**0.61**	0.58	0.59	0.49
Amphibians, Europe	p‐median	**0.55**	−0.18	−0.07	0.55	−0.18
	maxdisp	0.14	0.32	0.35	0.35	**0.71**
Reptiles, Europe	p‐median	0.56	0.38	**0.68**	0.29	−0.29
	**maxdisp**	0.62	**1.00**	0.57	0.42	0.42
Birds, Europe	p‐median	**0.36**	−0.04	0.09	−0.02	−0.14
	**maxdisp**	0.44	0.50	**0.69**	0.24	−0.06
Mammals, Europe	p‐median	−0.67	**1.00**	**1.00**	−0.67	−0.15
	maxdisp	−2.33	−0.25	**0.09**	−1.73	−2.33
Vertebrates, Europe	p‐median	**0.18**	0.08	−0.11	0.11	−0.23
	**maxdisp**	0.27	0.42	**0.47**	0.11	0.01

The best SAI value in each row is indicated in bold font, and the row median is underlined.

a Positive SAI values indicate the number of species represented in *p* sites selected by ED relative to the number of species represented in *p* randomly selected sites and the maximum number of species that could possibly be represented in *p* sites. Thus, SAI of 0.68 indicates that ED was 68% as effective as having full knowledge of true species distributions in its ability to improve on random selection of sites. Negative SAI values indicate that *p* sites selected by ED represented fewer species than p randomly selected sites.

The efficiency of both types of ED as a surrogate for species representation varied greatly across the five combinations of types of environmental variables used to define environmental space. Contrary to expectation, ordinations of environmental space using all five types of variables did not consistently produce higher (better) SAI values than ordinations using various subsets of environmental variables (Table 2). Ordinations using each of the five combinations of variable types was “best” for at least one dataset and ED approach, and there was no tendency for a particular combination of environmental variable types to work best for a particular taxon, scale, or cell size.

## 4 DISCUSSION

Beier and Albuquerque (2015) and Engelbrecht et al. (2016) provide strong evidence that ED is a reliable surrogate to represent plant and animal species. This study reinforces the finding of Engelbrecht et al. (2016) that maxdisp ED is as useful as the continuous p‐median ED, performing better than continuous p‐median for five of the 12 datasets we tested, arguably worse for one dataset (mammals of Europe), and about the same for the other six datasets. We believe that the strong performance of maxdisp relative to continuous p‐median is due to the fact that the POPSTAR implementation of the p‐median approach loses information about distances in environmental space when it collapses PCA space (up to eight dimensions) down to two dimensions. We chose POPSTAR because its heuristic consistently outperforms competing heuristics (Resende & Werneck, 2003; Mladenović, Brimberg, Hansen, & Moreno‐Pérez, 2007), but it is important to note that p‐median can be applied in any number of dimensions; indeed older software implemented the p‐median approach in six dimensions (Faith, Walker, Ive, & Belbin, 1996). But until future software implements POPSTAR's superior heuristic in multiple dimensions, maxdisp, by retaining information from all significant PCA axes, can better depict environmental space and select sites that span that space. In addition, for solutions involving more than a few thousand sites, computing time for continuous p‐median ED can take hours or days. Considering both the better performance of maxdisp and its much faster calculation (a few minutes), we agree with Engelbrecht et al. (2016) that maxdisp should be the method of choice in most applications of ED.

We acknowledge that continuous p‐median ED (unlike maxdisp) entails a clear theoretical expectation that selected sites will represent species efficiently (Faith & Walker, 1996). This potential advantage may be realized when software is developed to solve p‐median problems in 3–8 dimensions.

We were not surprised that the choice of type of environmental variables would affect the performance of both approaches to ED, but we expected that ordinations using all variables would consistently outperform ordinations using subsets of variables, as Engelbrecht et al. (2016) observed for six datasets in South Africa. This expectation was not supported by our evidence. We further expected that if differences across subsets of variables were large (as they were), then one or more subsets would be consistently bad (reflecting irrelevant or poorly measured variables) or that particular subsets (e.g., climate variables) would be superior for particular taxa (e.g., plants) or for coarser cell sizes (e.g., Europe versus Sierra Nevada or Arizona). This expectation was also not supported by our evidence. Clearly, additional investigations are needed to determine which environmental variables will produce superior ED models. Such investigations could improve on our effort by using >5 topographic variables, by including soil variables, and by testing across a greater variety of combinations of variables (e.g., combining different individual variables instead of broad types of variables). We suggest such tests should focus mostly on‐site sizes <10 × 10 km, because these small site sizes better match the parcel sizes at which conservation decisions are made.

About 80% of the ED models we tested, and about 83% of the best‐performing ED models, included climate variables, likely reflecting equilibrium of species’ distribution with contemporary climate. Species are said to be at equilibrium with climate if they occur in all climatically suitable areas while being absent from all unsuitable ones (*sensu* Araújo & Pearson, 2005). Many studies suggest that contemporary climate limits terrestrial taxonomic richness over broad geographic extents (Hawkins et al., 2003 and references therein). The climate hypothesis claims that energy availability and water‐energy dynamics generate and maintain richness patterns for most taxonomic groups (O'Brien, 1998; Hawkins et al., 2003). However, contemporary climate might or might not reflect future environmental diversity and species distributions (Wiens, Stralberg, Jongsomjit, Howell, & Snyder, 2009; Porfirio et al., 2014). The continuous p‐median ED approach may be appropriate for climate change impact assessment when climate variables are among the environmental variables used for deriving the space. Under climate change scenarios, demand points and species niches do not change (assuming no evolution in species niches), but the position of sites under climate change does change—producing a change in the p‐median that reflects gains and losses of species (Daniel Faith personal communication, June 10, 2017).

## CONFLICT OF INTEREST

None declared.
