# Supplementary material for: Improving the use of environmental diversity as a surrogate for species representation
Source: Ecol Evol. 2017 Dec 10;8(2):852–8. doi: 10.1002/ece3.3651 (PMC5773334; doi:10.1002/ece3.3651)
Supplement: Supplementary file 1 [file ECE3-8-852-s001.docx]

**Supplementary tables
Table S-1.** Predictors used as independent variables in models of continuous p-median and maxdisp environmental diversity. The combination of variable types is defined by a combination of environmental variables. CVT1 represents the combination of all variables, CVT2 comprises temperature, precipitation and vegetation variables, CVT3 represents temperature, precipitation and topography, CVT4 encompasses temperature and precipitation and CVT5 represents hours of sunshine and topography.

| **Temperature** | CVT1 | CVT2 | CVT3 | CVT4 | CVT5 |
| --- | --- | --- | --- | --- | --- |
| Annual mean temperature | x | x | x | x |  |
| Mean diurnal temperature range | x | x | x | x |  |
| Isothermality | x | x | x | x |  |
| Temperature seasonality | x | x | x | x |  |
| Max temperature of warmest month | x | x | x | x |  |
| Min temperature of coldest month | x | x | x | x |  |
| Annual temperature range | x | x | x | x |  |
| Mean temperature of wettest quarter | x | x | x | x |  |
| Mean temperature of driest quarter | x | x | x | x |  |
| Mean temperature of warmest quarter | x | x | x | x |  |
| Mean temperature of coldest quarter | x | x | x | x |  |
| **Precipitation** |  |  |  |  |  |
| Annual mean precipitation | x | x | x | x |  |
| Precipitation of wettest month | x | x | x | x |  |
| Precipitation of driest month | x | x | x | x |  |
| Precipitation seasonality | x | x | x | x |  |
| Precipitation of wettest quarter | x | x | x | x |  |
| Precipitation of driest quarter | x | x | x | x |  |
| Precipitation of warmest quarter | x | x | x | x |  |
| Precipitation of coldest quarter | x | x | x | x |  |
| **Insolation** |  |  |  |  |  |
| Hours of sunshine average | x |  |  |  | x |
| Hours of sunshine maximum | x |  |  |  | x |
| Hours of sunshine minimum | x |  |  |  | x |
| Hours of sunshine first quartile | x |  |  |  | x |
| Hours of sunshine fourth quartile | x |  |  |  | x |
| Hours of sunshine interquartile | x |  |  |  | x |
| Hours of sunshine range | x |  |  |  | x |
| **NDVI (normalized difference vegetation index)** | | | | |  |
| NDVI average | x | x |  | x |  |
| NDVI range | x | x |  | x |  |
| NDVI maximum | x | x |  | x |  |
| NDVI minimum | x | x |  | x |  |
| NDVI average | x | x |  | x |  |
| NDVI of the first quartile | x | x |  | x |  |
| NDVI of the fourth quartile | x | x |  | x |  |
| **Topography** |  |  |  |  |  |
| Range in elevation | x |  | x |  | x |
| Mean slope | x |  | x |  | x |
| Topographic aspect | x |  | x |  | x |
| Aspect diversity | x |  | x |  | x |
| Topographic diversity | x |  | x |  | x |

**Table S2**- Number of significant principal component analysis (PCA) factors for each of geographical areas in which environmental diversity was assessed.

| Combination of variable types | Sierra Nevada | Arizona | UK | Spain | Namibia | Bot-swana | Zim-babwe | Europe |
| --- | --- | --- | --- | --- | --- | --- | --- | --- |
| CVT1: all | 7 | 7 | 7 | 8 | 7 | 8 | 7 | 6 |
| CVT2: climate & NDVI | 5 | 5 | 5 | 6 | 5 | 3 | 5 | 5 |
| CVT3: climate, topography | 4 | 5 | 4 | 4 | 5 | 5 | 4 | 5 |
| CVT4: climate only | 2 | 3 | 3 | 4 | 4 | 3 | 4 | 4 |
| CVT5: insolation, topography | 4 | 3 | 4 | 3 | 4 | 4 | 3 | 3 |
